# Seeing by the Interior Sense

**Published:** 1887-08

**Authors:** 


### SEEING BY THE INTERIOR SENSE.

"Here is a man who is totally blind, but who nevertheless can see," said A. S. White, in introducing Henry Hendrickson to a visitor yesterday. And so it appeared. Mr. Hendrickson can see, or rather discern objects, although he was deprived of the sense of sight when he was six months old. He was born in Norway forty-three years ago, and has lived in America forty years. He was educated at the Institution for the Education of the Blind at Janesville, Wis., and has, since leaving that institution, followed various industries, notably that of broom-making, and is the author of a book entitled "Out from the Darkness." This work is somewhat in explanation of the second sight, with which he is becoming endowded, although he finds himself unable to account for it in any manner satisfactory to himself or conformable to physical science.

He is well educated, a somewhat brilliant conversationalist, and with glasses which hide his completely closed eyes, one would scarcely recognize him as a blind man. For the last twenty years he has seldom used an escort, except when in great haste and when going on territory entirely strange to him. It must be remembered that he is totally blind, and has never seen the light since he was six months old. Nevertheless, he can tell when he comes to a sudden rise in the sidewalk as well as one who enjoys complete sight; can turn a street corner, tell when he is passing an alley, approximate the height of the buildings along the street with apparent ease, but he cannot tell when he comes to a sudden depression in the sidewalk. For this he is unable to account. Many people who have observed the facility with which he moves from place to place, doubt that he is totally blind, but he has been put under the severest tests, and those who have made the investigations are convinced that he cannot see.

Yesterday, the *Herald* reporter spent some time with him at Mr. White's office, at 102 Washington street, and made a test of the blind man's wonderful second sight.

"When in a train at full speed," he said, "I can distinguish and count the telegraph poles easily, and often do it as a pastime or to determine our speed. Of course, I do not see them, but I perceive them. It is perception. Of course, my perceptive faculties are not in the least impaired on account of my blindness. I am not able to explain it, but I am never in total darkness. It is the same at midnight as at midday. There is always a bright glow of light surrounding me. Once, on being stung by a bee, I became for the moment stunned, and consequently blind, or, I should say, in total darkness. That is, I could not perceive or discern anything."

A practical test of this unaccountable second sight was made in the presence of the visitor. A thick, heavy cloth was thrown over his head as he sat in the chair. This hung down on all sides to his waist. It was impossible for any one to see through it. Then before him or behind him, it mattered not, an ordinary walking cane was held up in various positions. To such questions as: "Is it perpendicular or hori zontal?" or "In what position am I holding it?" he gave prompt and correct answers without a single mistake, sometimes describing acute or oblique angles. The test appeared so unaccountable that Mr. Hendrickson hastened to assure the guest that there was nothing supernatural about it. "It is wholly a matter of the perceptive powers," said the blind man, "But I cannot explain it further than that. Now, this covering is simply a formality; it is nonsense. I have never, by the ordinary sense of sight, seen an object in my life, not the faintest glimmer of one. My sight or discernment does not come in that way. This will prove the idea to you. Take me into a strange room, one that I have never been into and never heard about, and no matter how dark it is, I can tell you the dimensions of the room very closely. I do not feel the walls; I will touch nothing; I see nothing; but there is communicated to me by some strange law of perception, the size and configuration of the room. * * * I am studying short hand with Mr. White, and as my hearing is very good, I expect to become an expert. I had a little trouble with my writing at first—but am now able to write very well."

"Why, do you know," interjected Mr. White, "that when I stand up here in the room and with my projected forefinger make motions like one beating the time for a church choir, but describing phonetic characters, he can tell the characters I am making or describing, without seeing them, and can interpret them."

"Let us have a test on that line," requested the visitor.

With pleasure," responded Mr. Hendrickson with a smile. The guest further suggested that while he did not doubt Mr. Hendrickson's total blindness, he wished to have him blindfolded for the test.

"Certainly," said the blind man, and the robe was again brought into use. Then Mr. White stood up and cut the air rapidly, making certain phonetic characters.

"Well, you have asked me this," said Mr. Hendrickson, lifting the robe to get a breath of air, "Can you see what I am saying? I answer no and yes both. I don't see, but I know."

At this juncture the visitor bethought how the two might have put up a job or a joke upon him, and he suggested that he be allowed to write certain words upon a slip of paper, that Mr. White should repeat them phonetically by his forefinger, as before, and if then Mr. Hendrickson could tell what they were, blindfolded, as a mere matter of precaution, the proof would be conclusive.

"Let us have the test most certainly, and with pleasure," answered the blind man. The visitor wrote down the following upon a leaf from his note book, and passed it over to Mr. White.

"What are your politics?"

Mr. White struck off the question by ærial slants and curves and hooks. He had scarcely finished when Mr. H. slapped his hands with a laugh, and responded:

"Republican, of course."

"By the way," added Mr. Hendrickson, "I'm a very good skater, and can, when gliding over the ice swiftly, see every particle on the ice, every crack and rough spot, no matter how small or indistinct. The faster I go the plainer I can see. Well, I don't mean that I can see, but I perceive, or something. It is light to me, and I discern everything."

"Have you ever found yourself mistaken in depending upon this kind of sight?"

"Never. I was fooled once, but it came in this way: Once, when I was at Prairie du Chien, where I received a considerable sum of money for some six hundred dozen brooms which I sold, I got under the impression at night that I was being robbed. I saw the robber enter the bedroom door with a knife and a pistol. I laid quietly. He slipped his hand under the pillow, took the pocketbook and then ran out. I followed him and screamed. The house was immediately awakened. I said I had been robbed, but we could not find the robber. After breakfast it occurred to me that it was all a dream, and I returned to my room and found my pocketbook and the money where I left it."

Mr. Hendrickson is a wonderful man, and if his second sight is by some slight-of-hand art, it is very cleverly done.—*Chicago Herald.*

---

A writer in the British *Medical Journal* advises people to be careful not to slice up a pineapple with the same knife they use in peeling it, as the rind contains an acid organic substance, which is likely to cause a swollen mouth and sore lips. In Cuba salt is used as an antidote for the poison of pineapple peel.

